# A Preliminary Study of Hyperspectral Imaging Combined with Dual-Threshold Segmentation Technique for Peeling Rate Determination in Potatoes

**DOI:** 10.3390/s25247571

**Published:** 2025-12-13

**Authors:** Gaofeng Cao, Hongnan Hu, Qingyu Zhan, Tianwei Zhang, Yingbo Wang, Shiang Zhang, Lixue Zhu

**Affiliations:** 1 College of Mechanical and Electrical Engineering, Zhongkai University of Agriculture and Engineering, Guangzhou 510225, China; 17796560402@163.com (G.C.); zhanqingyu1116@163.com (Q.Z.); 18921668111@163.com (T.Z.); zhulixue@zhku.edu.cn (L.Z.); 2 College of Biosystems Engineering & Food Science, Zhejiang University, Hangzhou 310058, China; wangyb@hzcu.edu.cn; 3 School of Computer & Computing Science, Hangzhou City University, Hangzhou 310015, China; 4 College of Automation, Zhongkai University of Agriculture and Engineering, Guangzhou 510225, China; sazhang@zhku.edu.cn

**Keywords:** hyperspectral imaging, dual-threshold segmentation, peeling rate, potato

## Abstract

Peeling is essential in potato processing, yet conventional assessment methods face issues like inefficiency and environmental concerns. This study proposed a hyperspectral imaging approach combined with dual-threshold segmentation to quantify potato peeling rates. This method was in the preliminary research stage. The objectives of this work were to experimentally validate the feasibility and accuracy of this method. A hyperspectral system captured images of potatoes after water-jet peeling. By analyzing spectral data from peel and flesh regions and applying principal component analysis, the key wavelength of 592 ± 20 nm was identified, where the reflectance difference between flesh and peel was most pronounced. Grayscale images derived from this band were processed via median filtering and dual-threshold segmentation to differentiate flesh from peel. The peeling rate was calculated as the pixel ratio of flesh to total potato area. In validation tests, the calculated peeling rates showed an average absolute error of 0.69% compared to manual measurement, confirming the potential and feasibility of this technique. The proposed method offers a promising, non-destructive, and eco-friendly alternative for monitoring peeling quality in agricultural processing, though further research is warranted.

## 1. Introduction

Potato (*Solanum tuberosum* L.) is the fourth most important food crop globally, widely used in food processing, food production [1,2]. Peeling is one of the most important pretreatment steps in potato processing. Peeling directly determines raw material utilization efficiency, also critically influences product appearance and nutrient retention. Peeling rate is the core parameter for measuring peeling performance, an accurate and nondestructive peeling rate quantification method will enhance the mechanization level of peeling operations and play a significant guiding role in the optimization of peeling machinery. Traditional methods for determining the peeling rate mainly rely on manual measurement by calculating the ratio of the peeled surface area to the total surface area of the sample. However, manual measurement exhibits significant drawbacks, such as low efficiency and large errors [3]. To overcome the aforementioned problems, Li et al. [4]. proposed an iodine staining method to determine the potato peeling rate. This method used iodine to stain peeled potatoes, causing the starch on the surface to turn purple-black. The potato peeling rate was calculated by the ratio of the number of pixels in the stained area to the total number of pixels in the image. However, soaking peeled potatoes required a large amount of corrosive iodine solution and dyed potatoes could not be used, which will cause unnecessary waste and severe environmental pollution. So, it is necessary to come up with a new approach for potato peeling rate quantification through nondestructive technologies, which is precise, effective and environmentally friendly.

Hyperspectral imaging (HSI), a modern nondestructive detection technology, integrates imaging and spectral analysis to capture rich spatial and spectral information [5,6]. Compared with traditional RGB images, hyperspectral images can provide data of hundreds of continuous spectral bands, which can more accurately reflect the composition and property differences in samples [7,8]. This capability makes it particularly suitable for analyzing surface characteristics of agricultural products, where subtle differences in composition and morphology need to be identified. HSI technology has been widely applied in various fields, such as agriculture [9,10,11] and food industry [12,13]. Several studies have demonstrated the effectiveness of HSI in identifying surface features and defects in agricultural products, providing a solid foundation for our work. Zhao et al. [14] introduced a non-destructive detection method utilizing hyperspectral imaging combined with machine learning models, demonstrating the feasibility of identifying external defects in potatoes through this technology. Garhwal et al. [15]. devised a Zebra Chip (ZC) disease detection system utilizing HSI. They employed Partial Least Squares Discriminant Analysis (PLS-DA) models to identify ZC infected potatoes, achieving a recognition accuracy of 92%. Zhang et al. [16] integrated SPA, PCA, and chemical bond impact refine feature band selection for bruise diagnosis. They used the fast and precise YOLOv5 (FP-YOLOv5) model and the model achieved effective identification of apple bruises, with a high recognition rate of 95%. Pham and Liou [17] presented the development of an on-line surface defect system using hyperspectral images for jujubes. Support vector machine (SVM) and artificial neural networks (ANN) models were used to classify surface defects of jujubes. When the full wavelength range is used, the classification accuracy of ANN and SVM models for jujube peel defects are 96.5% and 96.3%, respectively. In order to improve the online detection efficiency, 14 characteristic bands were selected from 468~760 nm band by equal wavelength interval and principal component analysis (PCA). After optimization, the accuracy of SVM and Ann was 94.4% and 95% at equal interval 14 bands, and the accuracy of ANN and SVM was 95% and 94.6% at PCA 14 bands. Saeidan et al. [18] used PCA, support vector machine (SVM), linear discriminant analysis (LDA) and k-nearest neighbor (KNN) to analyze the spectral image data of 250 cocoa beans and foreign bodies. The accuracy of SVM in classifying cocoa beans and foreign bodies can exceed 89.10%. When the optimal feature was used as the input, the accuracy of SVM classifier was 86.90% on the training set and 81.28% on the test set. It can be seen that HSI technology has been successfully applied in quality inspection in agricultural products. However, the determination of peeling rate of agricultural products using HSI technology has not been widely adopted. Since hyperspectral images provide a large amount of spectral information, extract useful information from a vast amount of information is study-worthy. And the spectral reflectance of flesh of agricultural products differs significantly from that of the peel, HSI holds great potential for this specific segmentation task.

Identifying the peel and flesh of agricultural products is essential for calculating the peeling rate. To distinguish into multiple meaningful regions or segments, image segmentation methods are usually employed. More specifically, it assigns each pixel to a specific category or object based on various image features, such as pixel intensity, color, texture, position, or semantic information regarding the object’s category [19,20]. Image segmentation is widely used in various fields, such as digital image processing [21,22] and estimation of plant diseases [23,24]. Threshold segmentation is the most commonly used method in image segmentation. Cao et al. [25]. introduced a new method for extracting wheat lodging using a watershed algorithm integrated with an adaptive threshold segmentation hybrid algorithm, achieving an accuracy of 93.58%. Tian et al. [26]. developed a near-infrared (NIR) camera imaging technology combined with an adaptive threshold segmentation algorithm for early detection of apple bruises, with an accuracy of 95.56%. Zhou et al. [27]. designed an adaptive threshold segmentation algorithm based on the color space model to determined maize stem contours and diameters in the field, meeting the requirements for stem diameter measurement and providing a reference for acquiring maize phenotypic parameters in field conditions. These studies illustrate the widespread adoption of threshold segmentation methods. Overall, threshold segmentation can be used as a way to segment the peel and flesh of agricultural products.

This study proposed a novel, non-destructive approach that integrates hyperspectral imaging with a dual-threshold segmentation technique to quantify potato peeling rate. This method was in the preliminary research stage. The core innovative idea was to leverage the rich spectral information of HSI to identify a characteristic wavelength that distinguishes peel from flesh, and then to employ a dual-threshold segmentation algorithm to distinguishing between the background, potato flesh, and potato peel areas in grayscale images of the key characteristic bands, in order to calculate the potato peeling rate. This method was proposed as an alternative to conventional manual measurement, aiming to address issues of environmental impact and inefficiency. The objectives of this work were to experimentally validate the feasibility and accuracy of this integrated HSI and dual-threshold approach for peeling rate determination and to establish a foundation for the future development of non-destructive quality control in agricultural processing.

## 2. Materials and Methods

**Figure 1:**
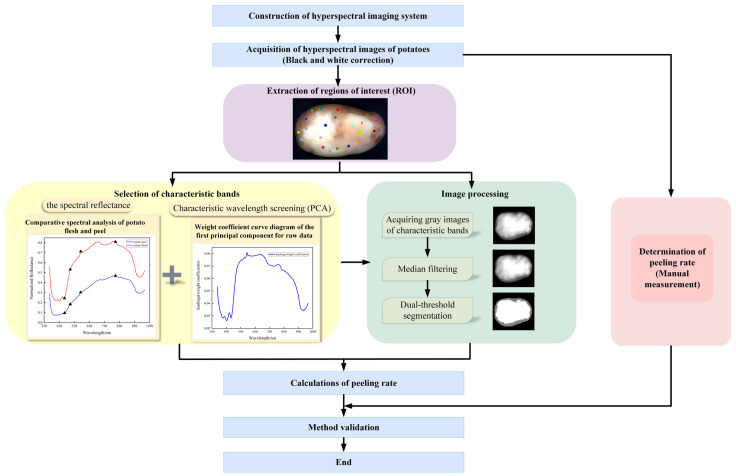
Work flow chart.

This study utilized a hyperspectral imaging system to acquire spectral images of potatoes following waterjet peeling. Spectral images were subjected to black and white correction, and regions of interest (ROIs) were selected to extract spectral information. Key feature bands were identified based on spectral curves and principal component analysis. Median filtering was applied to the grayscale images of these key bands, followed by dual-threshold segmentation to separate potato flesh and peel. The peeling rate was calculated as the pixel ratio between segmented potato flesh regions and potato regions. The flow is shown in Figure 1.

### 2.1. Samples Preparation

One hundred ‘Helan15’ potatoes were selected as test samples. All samples were measured three times with a vernier caliper, and the average values were recorded. The long axis of the samples was 93.6 mm and the short axis was 59.9 mm. Before image collection, all potatoes were peeled using the waterjet peeling machine (Guangzhou Linjun Mechanical & Electrical Equipment Co., Ltd., Guangzhou, China) and allowed to stand for 30 min under a ventilated indoor environment at 25 °C.

### 2.2. Hyperspectral Imaging System

**Figure 2:**
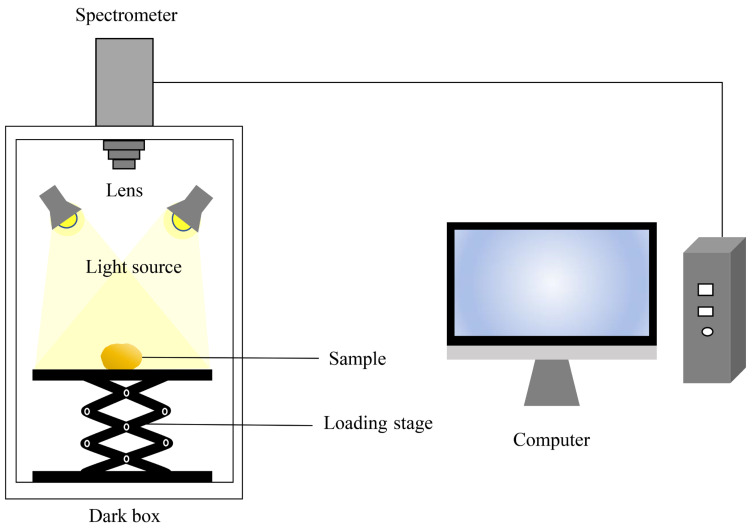
Hyperspectral imaging system.

In this experiment, the spectral image information of peeled potatoes was collected by using hyperspectral imaging system. This system consisted of a hyperspectral imager (model: GaiaSky-mini, Sichuan Dualix Spectral Imaging Technology Co., Ltd., Chengdu, China), a computer, an illumination system (four 50 W halogen lamps (Sichuan Dualix Spectral Imaging Technology Co., Ltd., Chengdu, China)), a sample stage, and a data acquisition dark box (Sichuan Dualix Spectral Imaging Technology Co., Ltd., Chengdu, China). The hyperspectral imager utilized a Sony ICX285 CCD sensor (Sichuan Dualix Spectral Imaging Technology Co., Ltd., Chengdu, China). To minimize the influence of ambient light, the experiment was conducted in a dark box. Prior to data acquisition, peeled potato samples were placed on a sample stage inside the box. Four 50 W halogen lamps were positioned symmetrically at the corners of the box, illuminating the samples at a 45° angle. The lens-to-sample distance was maintained at 48 cm, as shown in Figure 2.

Hyperspectral images of waterjet peeled potatoes were captured using the Gaiasky-mini’s integrated control software (version 2.9.1.5). The spectral range spanned 386.7–1016.7 nm, with image dimensions of 696 × 700 pixels. A total of 200 hyperspectral images were collected in this experiment.

The image data obtained by the hyperspectral imager is the relative value captured by the sensor, and data differences may occur due to different sensor types used by different devices. To eliminate these discrepancies, black and white correction is applied, converting relative reflectance into normalized reflectance, thereby ensuring data consistency and comparability [28]. The correction equation is:

(1) $$R=\frac{R_{0}-B}{W-B}$$

where *R* is the corrected image, *R*_0_ is the recorded hyperspectral image of the samples, *W* and *B* are the whiteboard image and blackboard image.

### 2.3. Selecting the Region of Interest (ROI)

**Figure 3:**
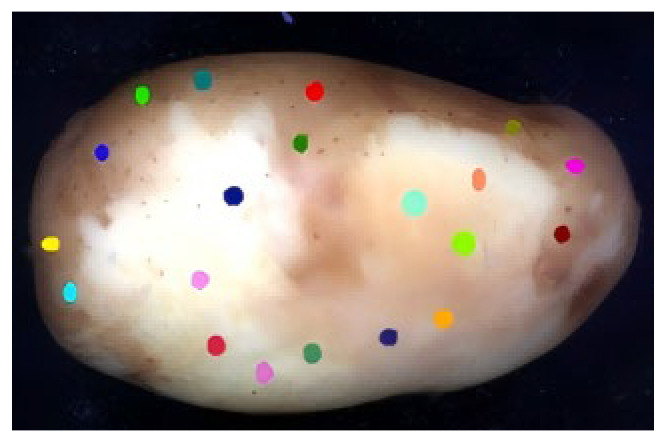
The selected regions of interest.

After acquiring hyperspectral images of peeled potatoes, ENVI software (version 5.6) was used in this study to extract spectral curve data. In order to effectively extract the characteristic spectra of potato peel and flesh, two types of ROIs were selected in this study: potato peel and flesh (internal tuber). 20 ROIs (10 peel regions and 10 flesh regions) were extracted from each image. Figure 3 indicates the selected regions of interest.

### 2.4. Principal Component Analysis (PCA)

PCA is an unsupervised dimensionality reduction technique that aims to map high-dimensional data to a lower-dimensional space through linear transformation while maximizing the preservation of variance information in the original data. The core principle involves extracting the most representative principal components, which are new variables that are mutually orthogonal and composed of linear combinations of the original features. These principal components are arranged in descending order of their variance contribution. By retaining the top few principal components with the greatest variance, PCA can effectively remove noise and redundant information while reducing data dimensionality and simplifying the computational complexity [29].

### 2.5. Image Preprocessing

Preprocessing is a critical step in image analysis and processing, as it can significantly enhance the accuracy and stability of subsequent processing. In this study, grayscale images of potatoes were subjected to various noises, including random noise from imaging equipment and external factors during acquisition. To mitigate these effects on subsequent analysis, a median filtering method was employed to smooth the images.

Median filtering, a nonlinear technique based on ranking statistics, is extensively employed in image denoising, particularly effective for eliminating impulse noise. By substituting each pixel value with the median of its neighborhood, it preserves edge information while reducing noise. The core idea is to leverage the distributional characteristics of pixel values in the local vicinity to suppress the randomness of noise while avoiding excessive blurring of the image’s structural features. The equation is

(2) $$I_{denoised}(x,y)=Median\{I(x+1,y+1)|-2\leq i,j\leq 2\}$$

where *I*(*x*,*y*) denotes the pixel value at position (*x*,*y*) of the original image, and *I_denoised_* (*x*, *y*) represents the pixel value after median filtering.

### 2.6. Dual-Threshold Segmentation

Image segmentation represents a critical step in image processing, this study aimed to distinguish potato peel, flesh, and background regions. However, a single-threshold segmentation method struggles to deal with the segmentation problems of potato peel and flesh regions simultaneously. To address this problem, a dual-threshold segmentation approach was adopted. By defining two distinct thresholds, the image was partitioned into three different regions: background, potato peel, and potato flesh. This method could handle complex lighting conditions and noise interference more effectively, thereby enhancing segmentation accuracy and reliability.

Two thresholds were defined: a low threshold (*T_low_*) and a high threshold (*T_high_*). The selection of these two thresholds was based on the analysis of the grayscale image and the observed gray characteristics of potato peel and flesh. *T_low_* was used to distinguish the background and potato area, while *T_high_* differentiated between potato peel and potato flesh. Through these two thresholds, pixels in the image were classified into the following three regions: (1) Background area, where pixel values fell below *T_low_*, indicating non-potato areas. (2) Potato peel area, with pixel values between *T_low_* and *T_high_*. (3) Potato flesh area, where pixel values exceeded *T_high_*. In order to achieve the above classification, two masks were created: the potato peel mask (*M_peel_*) and the potato flesh mask (*M_flesh_*), generated by comparing pixel values against the thresholds. The equation is:

(3) $$M_{peel}(x,y)=\left\{\begin{array}{l} 1,\;if\;T_{low\;}\leq I_{denoised}(x,y)<T_{high}\\ 0,\;otherwise\; \end{array}\right.$$

(4) $$M_{flesh}(x,y)=\left\{\begin{array}{l} 1,\;if\;I_{demoised}(x,y)\geq T_{high}\\ 0,\;otherwise\; \end{array}\right.$$

where *I_denoised_* (*x*, *y*) represents the pixel value of the image after median filtering at the position (*x*, *y*).

Using these two masks, the pixels in the potato peel and potato flesh regions could be extracted, respectively, providing a basis for further analysis. After the masks were generated, the number of pixels in the potato peel and potato flesh regions was counted. The equation is:

(5) $$N_{peel}=\sum_{x,y}M_{peel}(x,y)$$

(6) $$N_{flesh}=\sum_{x,y}M_{flesh}(x,y)$$

(7) $$Q=N_{peel}+N_{flesh}$$

where *N_peel_* is the number of pixels in potato peel area, *N_flesh_* is the number of pixels in potato flesh area and the total number of potato pixels *Q* is the sum of the two.

### 2.7. Calculating the Potato Peeling Rate

The peeling rate serves as a crucial metric for evaluating the efficiency and quality of potato peeling. In this study, the peeling rate was defined as the ratio of the number of pixel points in the potato flesh area to the total number of pixel points in the potato. The equation is

(8) $$C=\frac{N_{flesh-front}+N_{flesh-back}}{Q_{front}+Q_{back}}\times 100\%$$

where *N_flesh-front_* is the number of pixel points in the front of potato flesh, *N_flesh-back_* is the number of pixel points on the back of potato flesh, *Q_front_* is the total number of pixel points in the front of potato, *Q_back_* is the total number of pixel points on the back of potato, *C* is the peeling rate.

In order to verify the accuracy of the method, a comparative test was conducted. In the experiment, actual peeling rate was determined by manual measurement. This method provides a reliable comparison benchmark for this study.

## 3. Results

### 3.1. Spectral Analysis

**Figure 4:**
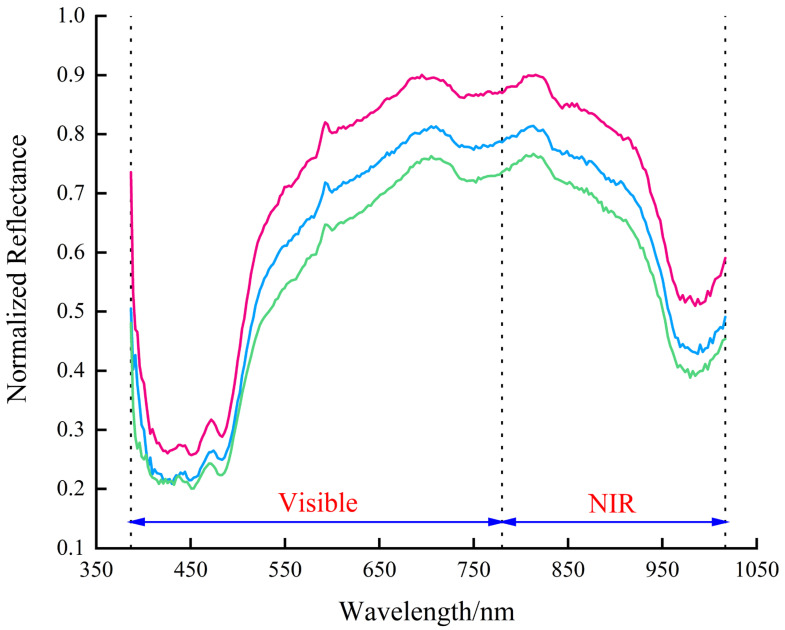
Representative hyperspectral reflectance curves of potato flesh. Three individual curves are shown to illustrate the characteristic spectral profile and the range of natural variation observed across different flesh regions. The azure, magenta, and cyan curves represent the most common, a higher, and a lower reflectance profile, respectively.

**Figure 5:**
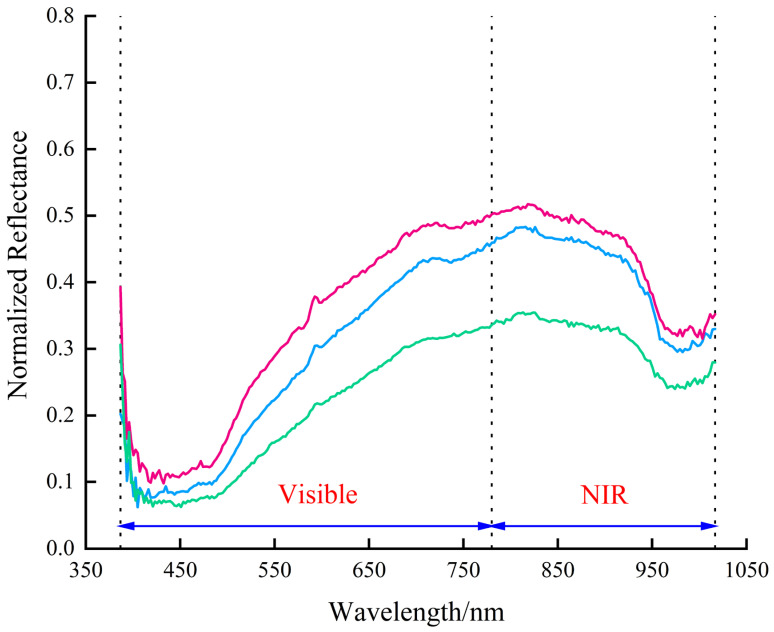
Representative hyperspectral reflectance curves of potato peel. Three individual curves are shown to illustrate the characteristic spectral profile and the range of natural variation observed across different peel regions. The azure, magenta, and cyan curves represent the most common, a higher, and a lower reflectance profile, respectively.

This study focused on two regions of interest: potato peel and flesh. Following spectral extraction and black-and-white correction, reflectivity data were plotted in the same figure. Representative reflectance curves from multiple regions of interest (ROIs) for potato flesh and peel are presented in Figure 4 and Figure 5, respectively. Each figure displays three selected curves that illustrate the characteristic spectral trend and the natural range of reflectance variation within each category.

The figures illustrate notable differences in the spectral reflectance curves of potato flesh and peel, with the flesh generally exhibiting higher reflectance. This discrepancy primarily arose from the peel’s color variability, where darker areas led to reduced reflectance. After waterjet peeling, the color of potato flesh varied slightly due to differences in peeling loss rates. Nevertheless, the overall trends of spectral curves for both remained similar.

**Figure 6:**
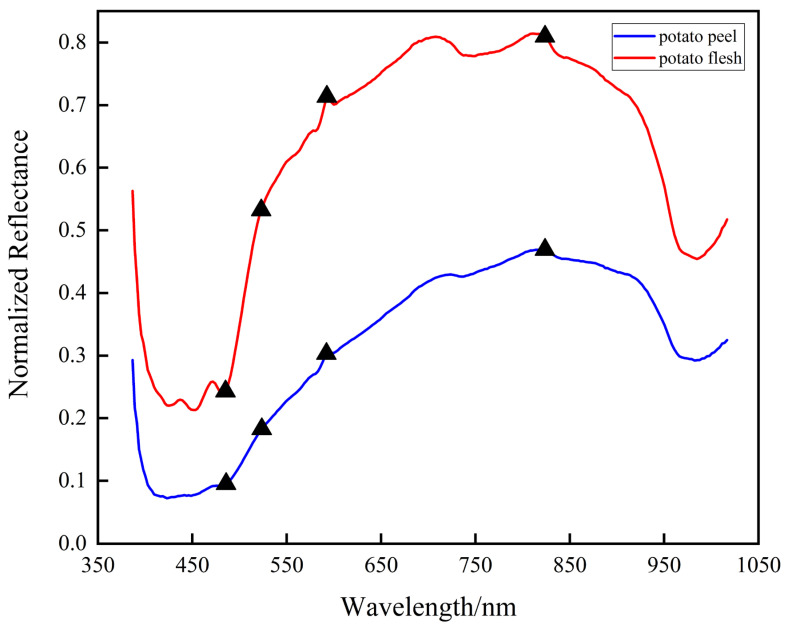
Comparative spectral analysis of potato flesh and peel.

To more clearly demonstrate the spectral reflectance differences between potato flesh and peel, we averaged spectral data sets for each category and plotted the results in Figure 6.

From the 485 nm band, both potato flesh and peel exhibited an upward trend in reflectance, but the increase was more pronounced in potato flesh. In the 485–523 nm wavelength range, the reflectance slope of potato flesh was significantly steeper than that of potato peel, indicating a more rapid increase in the light reflectance capacity of potato flesh in this band. Within the 592–1016.7 nm range, the trends of two curves converged without obvious slope differences, though the reflectance of potato flesh remained higher than potato peel. Starting from the 823 nm band, the reflectance of both potato flesh and peel showed a gradual downward trend.

### 3.2. Result Analysis Using PCA Method

**Figure 7:**
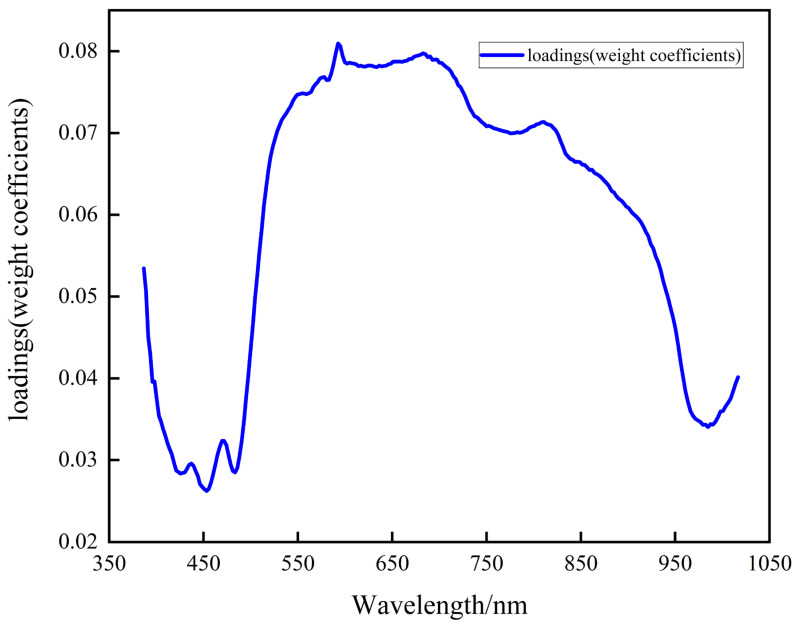
Weight coefficient curve diagram of the first principal component for raw data.

The input data consisted of the samples extracted from the region of interest, each with 256 dimensions. We applied PCA to reduce the dimensionality to 5. After dimensionality reduction, the contribution rates of the five principal components were as follows: PC1 = 98.16%, PC2 = 1.08%, PC3 = 0.49%, PC4 = 0.10%, and PC5 = 0.05%. The first principal component contributed the most (98.16%), while the fifth contributed the least (0.05%). Since the proportion of the first principal component exceeded 90%, it could completely substitute the original data. Figure 7 demonstrates the distribution of weight coefficients for 256-dimensional data under the first principal component.

The figure reveals that the peak value of the weight coefficient of the first principal component occurred at 592 ± 20 nm, with an average value of 0.081. This indicated that this band significantly contributed to the first principal component and effectively differentiated between potato peel and flesh.

### 3.3. Selection of Characteristic Bands

Selecting the appropriate spectral band is crucial for enhancing the accuracy and computational efficiency in calculating the potato peeling rate. Spectral reflectance curves of potato flesh and peel were analyzed following black and white correction to identify bands exhibiting significant reflectance differences between the two, which were then utilized for subsequent peeling rate calculations. The analysis revealed that beginning at the 485 nm wavelength, both potato flesh and peel exhibited increasing reflectance, with the flesh showing a more pronounced rise. In the 485–523 nm band range, the reflectance slope of potato flesh exceeded that of potato peel, demonstrating a more rapid increase in the flesh’s light reflection within this spectrum. Within the 592–1016.7 nm range, potato flesh consistently exhibited higher reflectivity than potato peel, although both followed similar trends without significant differences in slope. From 823 nm onward, the reflectance of both flesh and peel gradually decreased.

Combined with PCA, the rationality of band selection was further verified. The analysis revealed that the highest weight coefficient in the first principal component was within the 592 ± 20 nm range, with an average weight coefficient of 0.081 in this range. This suggested that this band range made a greater contribution to the first principal component, and the spectral information within this band range could effectively distinguish potato peel and potato flesh.

Based on the spectral reflectance curve analysis and the PCA results, the 592 ± 20 nm band was selected as the key band for the subsequent calculation of potato peeling rate. This band demonstrated remarkable discrimination ability in spectral reflection characteristics and contributed heavily in PCA, offering precise and efficient support for calculating the peeling rate.

### 3.4. Median Filtering and Dual-Threshold Segmentation Analysis

**Figure 8:**
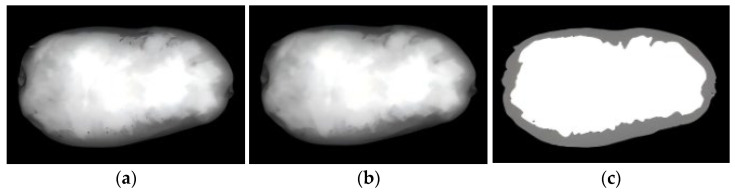
Image processing workflow: from original image to denoising and segmentation: (**a**) Original image; (**b**) Denoised image after median filtering; (**c**) The effect of using dual-threshold segmentation method on denoised image.

Grayscale images of peeled potatoes in the 592 ± 20 nm wavelength band were processed using a Python program (version 3.10.16). A 5 × 5 median filter was applied to these images, with two thresholds set to differentiate among the potato peel, potato flesh, and background: *T_low_* = 35, *T_high_* = 125. By adjusting and narrowing threshold ranges, we observed the segmentation effect of potato peel, potato flesh, and background. We found that setting *T_low_* at 35 and *T_high_* at 125 effectively separated the peel and flesh regions. Figure 8 reveals the effect after median filtering and dual-threshold segmentation.

Image processing and visual analysis were performed using the program, which effectively suppressed image noise while preserving edge and texture information. This process enabled the segmentation of potato flesh, peel, and background regions, validating the appropriate selection of characteristic bands and providing a basis for subsequent peeling rate calculation.

### 3.5. Calculations of Peeling Rate

**Figure 9:**
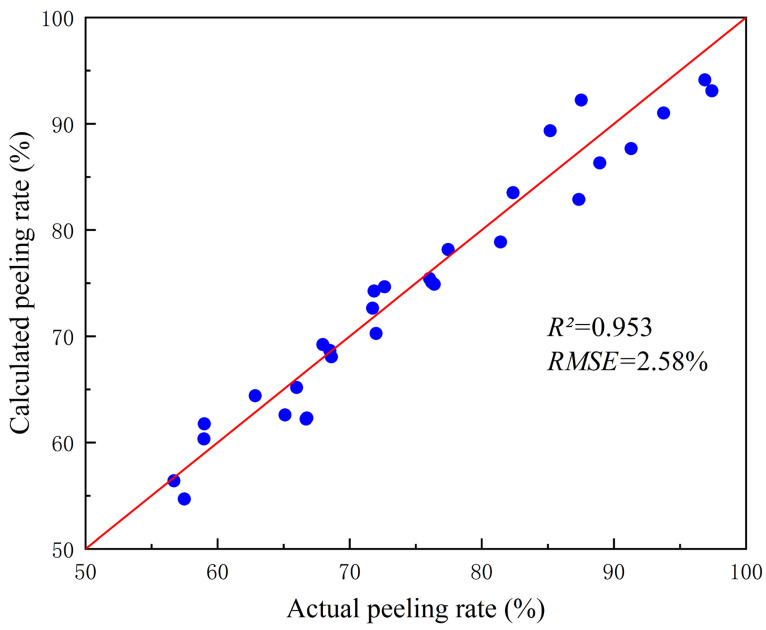
Results of calculated peeling rate and actual peeling rate.

Thirty potato samples were randomly selected for the experiment. Following median filtering and dual-threshold segmentation, potato peeling rate was calculated according to the above Equation (8). Figure 9 presents the calculation results of peeling rates for different potato samples, including the calculated peeling rates, the actual peeling rates determining through manual measurement.

The results presented in Figure 9 indicated that the proposed method achieved high accuracy in calculating the potato peeling rate. The average calculated peeling rate was 74.34%, closely aligning with the 75.04% average obtained using the manual measurement, resulting in a mere 0.69% average error. This small error demonstrated the method’s reliability and accuracy. Furthermore, the standard deviation of the error was 2.58%, indicating that the difference between the calculated peeling rate and the actual peeling rate had high consistency and stability among different samples. Even if there were individual differences in the samples, the method maintained stable computational accuracy without significant error fluctuations. These data further verified the effectiveness and reliability of this method.

## 4. Discussion

The present study served as a feasibility assessment for determining potato peeling rates using a hyperspectral imaging approach combined with a dual-threshold segmentation algorithm. Our findings validated the core premise: by identifying key characteristic bands through PCA of spectral data from peel and flesh, and subsequently processing the corresponding grayscale images with median filtering and dual-threshold segmentation, it was possible to effectively differentiate between these regions. This successful discrimination forms the basis for a novel, non-destructive method to calculate peeling rate, confirming the initial feasibility of the proposed technique.

In comparison to manual measurement, the proposed method presents itself as a potential alternative that could help mitigate issues of subjectivity and efficiency. The experimental results, showing a small mean error (0.69%) and consistent performance across samples (standard deviation of 2.58%), indicate its practical feasibility. This initial success lays the groundwork for future research to explore its full potential in industrial applications.

**Figure 10:**
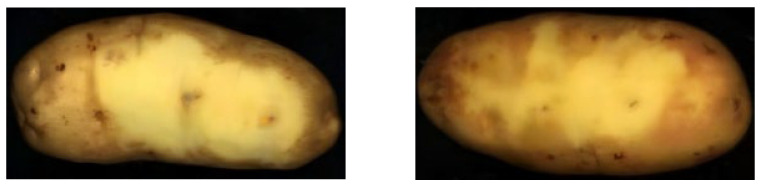
This figure shows that some areas of the potato are difficult to distinguish between the peel and the flesh after the potato has been peeled by the waterjet.

**Figure 11:**
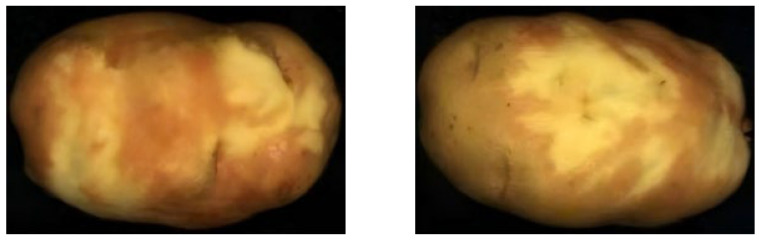
This figure displays that the peeled areas of potatoes exhibit some color changes after being peeled by waterjet and left for a period of time.

This study found that after the potatoes are peeled by waterjet, sometimes it is difficult to distinguish between potato peel and flesh in some areas, which will result in variable reflectance spectra for selected regions of interest, as shown in Figure 10. Additionally, peeled potatoes to sit for a period induces color changes, further altering the spectral curve reflectance in some regions of interest, as shown in Figure 11. Therefore, when selecting the region of interest, we should select the region that can distinguish the potato peel and flesh.

Therefore, when selecting the region of interest, we should select the region that can clearly distinguish the potato peel and flesh. Although this study successfully demonstrated the feasibility of the proposed method under controlled laboratory conditions, it is important to acknowledge its limitations. Most notably, the dual thresholds (*T_low_* = 35 and *T_high_* = 125) used in this method to distinguish the background, peel, and flesh were fixed values determined from a specific dataset. These threshold parameters are not universal constants. They are likely to vary with differences in imaging systems (e.g., camera model, light source intensity and angle), environmental conditions, and the inherent characteristics of the potato varieties themselves. This dependency on specific experimental conditions limits the ability of the current model to be directly applied to broader industrial scenarios without calibration. However, this very limitation defines the most promising direction for future research. To address this issue and enhance the robustness and practicality of the method, future work will focus on developing machine learning or developing algorithms capable of determining these thresholds adaptively. There are some limitations because it is not compared with other segmentation techniques. We will also systematically compare different segmentation algorithms to find the optimal one.

However, the reliability and applicability of method or model will be reduced due to different varieties [30,31]. Different varieties of potatoes, due to the differences in their own material composition and peel characteristics, result in different spectral image features, making it difficult for the selected thresholds to adapt to all varieties. Therefore, in future work, more attempts will be applied in determining peeling rate by using other types of potatoes. And stage of maturity, moisture content, and post-harvest handling can significantly influence spectral characteristics. For the development of a robust, generalized model with true industrial applicability, it is essential to account for this natural variability. Therefore, building upon the promising results of this preliminary study, our future work will focus on a comprehensive validation and scaling effort. The experimental design will systematically encompass multiple commercial potato varieties, different maturity stages, and a range of storage conditions. The objective of this subsequent research is to develop a stable, generalizable, and practical system that can reliably monitor peeling quality across the diverse raw materials encountered in industrial potato processing.

## 5. Conclusions

This study preliminarily investigated the feasibility of a dual-threshold segmentation method based on hyperspectral imaging for determining the potato peeling rate. The key characteristic band at 592 ± 20 nm was identified through analysis of spectral reflectance curves and PCA, effectively distinguishing between potato flesh and peel. By applying median filtering and dual-threshold segmentation (*T_low_* = 35, *T_high_* = 125) to grayscale images within this band, the peeling rate was computed. The close agreement between the calculated average peeling rate (74.34%) and the manual measurement result (75.04%), with a minor average discrepancy of 0.69%, demonstrates the potential feasibility and reliability of the proposed approach. These findings suggest that hyperspectral imaging combined with dual-threshold segmentation can serve as a promising alternative for non-destructive peeling rate assessment. As a preliminary study, this work lays a foundation for future research. Further validation should include expanding the dataset, testing on a wider range of potato varieties, and exploring more advanced image processing algorithms to enhance robustness and adaptability across different industrial conditions. The method’s potential application to other root vegetables also warrants investigation.

## Data Availability

The original contributions presented in this study are included in this article. Further inquiries can be directed to the corresponding author.

## Abbreviations

The following abbreviations are used in this manuscript:

PCA	Principal Component Analysis
ROI	Region of Interest
HSI	Hyperspectral imaging
SSC	Soluble Solid Content
ZC	Zebra Chip
PLS-DA	Partial Least Squares Discriminant Analysis
PLSR	Partial Least Squares Regression
SMV	Soybean Mosaic Virus
CNN	Convolutional Neural Networks
SVM	Support Vector Machines
NIR	Near-infrared
